# A Red-Berry Mixture as a Nutraceutical: Detailed Composition and Neuronal Protective Effect

**DOI:** 10.3390/molecules26113210

**Published:** 2021-05-27

**Authors:** Noelia Carballeda-Sangiao, Susana Chamorro, Sonia de Pascual-Teresa

**Affiliations:** 1 Departamento de Metabolismo y Nutrición, Instituto de Ciencia y Tecnología de Alimentos y Nutrición (ICTAN-CSIC), C/José Antonio Nováis, 10, 28040 Madrid, Spain; n.carballeda@ictan.csic.es (N.C.-S.); schamorr@ucm.es (S.C.); 2 Departamento de Genética, Fisiología y Microbiología, Facultad de Ciencias Biológicas, Universidad Complutense de Madrid (UCM), 28040 Madrid, Spain

**Keywords:** polyphenol, anthocyanin, neuronal, antioxidant, red-berry

## Abstract

Recommendations towards increased consumption of fresh fruit and vegetables are well supported by epidemiological and clinical trials. However, in some specific cases, it is difficult to follow these recommendations and the use of nutraceuticals or, in the present work, a freeze-dried fruits mixture can be recommended in order to afford the optimal consumption of dietary polyphenols naturally present in fruits and vegetables. In this work we have carefully characterized a red-berry mixture in terms of polyphenol composition, encountering mainly anthocyanins, which account for a total of 2.8 mg/g as cyanidin-3-glucoside equivalents. Additionally, we have assayed the red-berry blend in a cell model of neurological damage by differentiating the cells and measuring the effect of red-berry polyphenols on cell viability and redox state by flow cytometry. The berry-fruit extract showed an inhibitory effect on differentiated SH-SY5Y ROS formation at a concentration as low as 250 µg/mL (33% inhibition). The results show the potential of this berry-fruit blend for its nutraceutical use in the prevention of the neurodegeneration associated with age or environmental agents.

## 1. Introduction

Aging under normal physiological conditions leads to impaired cognitive function. This age-related cognitive decline causes difficulties with memory, executive function, and processing speed, which can begin as early as the fourth decade of life [1,2,3]. The etiology of these processes is not well understood, although it is assumed that it is linked to an increase in oxidative stress and a reduction in the function of the immune system. Scientific findings in recent years have contributed to better understand the interrelationship between aging, cardiovascular disease, insulin resistance, obesity, inflammation and, more recently, the microbiota [4,5]. However, the damage associated with aging is multifactorial and lifestyle habits, including diet and physical exercise, play a fundamental role in promoting health, preventing it and ultimately improving the well-being of the general society. In this sense, numerous epidemiological and clinical studies suggest that there is a positive association between the intake of polyphenol-rich extracts and the improvement of cognitive performance and, in particular, working memory and attention during a high-effort cognitive challenge [3,6,7]

Anthocyanins are natural pigments that are present in various fruits and vegetables. Red fruits and young red wines are especially rich in this group of flavonoids. The most common anthocyanidins are cyanidin, pelargonidin, peonidin, delphinidin, petunidin, and malvidin. These anthocyanidins, when glycosylated in different positions, give rise to anthocyanins [8]. Flavanols are also flavonoid polyphenols, and include monomeric compounds, also called catechins (catechin and epicatechin), and others polymerized with each other, also called proanthocyanidins. They are compounds that are characterized by the C6-C3-C6 structure, for being colorless (in contrast to anthocyanins) and for being widely distributed in fruits, cereals and vegetables, although fruits are the main source of flavanols in the diet.

Different authors have demonstrated the ability of some polyphenols, such as (+)–catechin, (−)–epicatechin [9,10] and cyanidin-3-rutinoside [11], to cross the blood–brain barrier (BBB) and reach the brain tissues independently of their route of administration. The pharmacokinetics of (+)-catechin and (−)-epicatechin and their ability to cross the BBB, entering into the extracellular fluid of the hippocampus, has been reported in rats by microdialysis [12]. Additionally, the presence of several anthocyanins (among them cyanidin-3-*O*-glucoside and galactoside, peonidin-3-*O*-arabinose and delphinidin-3-galactosides) has been observed in the brain of rats fed blueberries [13] and, after their intravenous administration [14], subtending the capacity of these compounds to affect learning activity and memory. The intake of berries ameliorated several aspects of memory and learning, and delayed age-related motor and cognitive behavioral deficits in rodent models [15]. In addition to the experimental evidence, the neuroprotective potential of polyphenols and their derived metabolites to inhibit ROS generation and protect neuronal cells from oxidative damage or death has been studied in culture models [16,17]. These models allow the study of the mechanisms involved in the potential neuroprotective effect of dietary polyphenols and the interactions between both groups of flavonoids at a mechanistic level, thus allowing the future optimization of the design of diets or foods enriched in both groups of bioactive compounds aimed at a defined sector of the population [17,18].

The aim of the present study was to determine the detailed polyphenolic composition of a red-berry mixture extract (RB) using high performance liquid chromatography coupled with high resolution Q-TOF mass spectrometry (HPLC-MS-QTOF), and to evaluate its antioxidant and neuroprotective effects on a human neuronal cell model.

## 2. Results

### 2.1. Anthocyanins

The analysis of anthocyanins in the red-berry extract was carried out by means of HPLC-QTOF-MS analysis as it is detailed in the methodology section. Characterization of the anthocyanins presented in the berry extract is reported in Table 1.

Cyanidin derivatives were identified based on the presence of their characteristic aglycon fragment at *m/z* 287. Among them, the first molecular ion at *m/z* 449 produced an MSMS fragment at *m/z* 287 which corresponded to the loss of a glucose (*m/z* 162) and its identity was confirmed with its corresponding standard. A second ion at *m/z* 595 produced MSMS fragments at *m/z* 287 and 449 and was identified as cyanidin-3-O-rutinoside in accordance with published results [19]. The third ion at *m/z* 419 produced an MSMS fragmentation at 287 which corresponded with a loss of arabinose (*m/z* 132) and was identified as Cyanidin-3-O-arabinoside according to our previous results [20].

Delphinidin derivatives were identified based on the presence of their characteristic aglycon fragment at *m/z* 303. The first ion at *m/z* 465 showed in the MSMS spectra the loss of a glucose (*m/z* 162) and was identified as delphinidin-3-O-glucoside. A second ion at *m/z* 611 producing MSMS fragments at *m/z* 465 and 303 was identified as delphinidin-3-O-glucoside in accordance with the scientific literature [19].

Peonidin derivatives were identified based on the presence of their characteristic aglycon fragment at *m/z* 301. Among them, the first molecular ion with *m/z* at 463 corresponding to the loss of a glucose was identified as peonidin-3-O-glucoside and was confirmed by comparison with its commercial standard. A second ion at *m/z* 609 and a fragmentation pattern at *m/z* 463 and 301 was identified as peonidin-3-O-rutinoside, whereas a third ion at 433 yielding a MSMS fragment at *m/z* 301, which corresponded to a loss of an arabinose (*m/z* 132), was identified as peonidin-3-O-arabinoside.

### 2.2. Flavonols, Flavanols and Free Aminoacids

The characterization of flavonols was performed based on the presence of their aglycon myricetin (*m/z* 319), quercetin (*m/z* 303) and kaempferol (*m/z* 287) derived from the losses of a hexose, either glucose or galactose (*m/z* 162), a pentose such as arabinose or xylose (*m/z* 132), rhamnose (*m/z* 146), rutinose (*m/z* 308) or a glucuronide (*m/z* 176) unit during the MSMS fragmentation. Glycosylation positions were tentatively proposed by considering the relative abundance of the aglycone fragment during mass fragmentation according with the literature and our own experience with the identification of such isomers [21,22].

**Table 1:** Characterization of polyphenolic compounds and free amino acids in the red-berry extract.

Compound Assignment	RT ^1^ (min)	Molecular Formula	[M + H]^+^ Theoretical	[M + H] Identified	MS/MS	Identification ^2^	Content mg/100 g
*Anthocyanins*							
Delphinidin-3-*O*-glucoside	7.9	C_21_H_21_O_12_	465.1028	465.1042	303	MS/MS (1)	37.4 ± 2.3
Delphinidin-3-*O*-rutinoside	8.6	C_27_H_31_O_16_	611.1607	611.1610	303/465	MS/MS (2)	89.4 ± 7.6
Cyanidin-3-*O*-glucoside	9.5	C_21_H_21_O_11_	449.1083	449,1089	287	Std	28.2 ± 2.9
Cyanidin-3-*O*- rutinoside	10.5	C_27_H_31_O_15_	595.1657	595.1661	287/449	MS/MS (2)	69.9 ± 5.5
Cyanidin 3-*O*-arabinoside	11	C_20_H_19_O_10_	419.0973	419.0977	287	MS/MS (1)	3.53 ± 0.4
Peonidin-3-*O*-glucoside	12.3	C_22_H_23_O_11_	463.1243	463.1235	301	Std	33.3 ± 2.8
Peonidin 3-O-rutinoside	13.6	C_28_H_33_O_15_	609.1819	609.1820	301/463	MS/MS (1)	4.46 ± 0.2
Peonidin-3-*O*-arabinoside	14.2	C_21_H_21_O_10_	433.1141	433.1129	301	MS/MS (3)	11.5 ± 1.0
*Flavonols*							
Myricetin-3-*O*-rutinoside	18.8	C_27_H_30_O_17_	627.1556	627.1560	319/481	MS/MS (4)	9.12 ± 0.7
Myricetin-3-*O*- galactoside	19.2	C_21_H_20_O_13_	481.0977	481.0961	319	MS/MS (4)	7.78 ± 1.3
Myricetin-3-*O*- glucoside	19.8	C_21_H_20_O_13_	481.0977	481.0983	319	MS/MS	7.38 ± 1.5
Quercetin-3-*O* rutinoside	22.1	C_27_H_31_O_16_	611.1607	611.1599	303	MS/MS (4)	5.73 ± 1.2
Quercetin-3-*O*-galactoside	23.2	C_21_H_20_O_12_	465.1028	465.1035	303	MS/MS (5)	10.9 ± 2.1
Quercetin-3-*O*-glucoside	23.6	C_21_H_20_O_12_	465.1028	465.1042	303	Std	3.18 ± 0.5
Quercetin-3-*O*-glucuronide	24.1	C_21_H_18_O_13_	479.0820	479.0836	303	MS/MS	0.67 ± 0.4
Quercetin pentoside	25.2	C_20_H_18_O_11_	435.0922	435.0937	303	MS/MS	0.95 ± 0.3
Quercetin pentoside	25.9	C_20_H_18_O_11_	435.0922	435.0934	303	MS/MS	0.93 ± 0.3
Quercetin pentoside	26.6	C_20_H_18_O_11_	435.0922	435.0926	303	MS/MS	0.62 ± 0.2
Kaempferol-3-*O*-glucoside	27.0	C_21_H_20_O_11_	449.109	449.1078	287	MS/MS	0.90 ± 0.2
Quercetin	38.3	C_15_H_10_O_7_	303.0499	303.0512	.	Std	1.18 ± 0.4
*Flavan-3-ols*							
Epicatechin	15.8	C_15_H_14_O_6_	291.0863	291.0880	121/139	Std	3.84 ± 0.8
*Amino acids*							
l- phenylalanine	5.2	C_9_H_11_NO_2_	166.0863	166.0861	120	Std	3.89 ± 0.4
l- tryptophan	8.3	C_11_H_12_N_2_O_2_	205.0976	205.0980	146	Std	1.28 ± 0.2

^1^ RT, retention time. ^2^ Identification was confirmed according to the standard (Std) and/or MSn fragmentation pattern previously described by other authors: (1) [20]; (2) [19]; (3) [23]; (4) [24]; (5) [25].

In this sense, the prevalence of the aglycone radical during MSMS fragmentation was indicative of 3-*O*-glycosyde in our conditions. Three peaks releasing MS fragments at *m/z* 317, were identified as myricetin derivatives. The first peak with an *m/z* 627 produced two MSMS fragments (at *m/z* 481 and 319) and was identified as myricetin-3-*O*-rutinoside. Another two peaks with a similar molecular ion at *m/z* 481 and releasing an MSMS fragment at *m/z* 319 were differentiated according to their elution order and were identified as myricetin-3-*O*-galactoside and myricetin-3-*O*-glucoside, respectively. Seven peaks releasing a unique MS fragment at *m/z* 303 were assigned as quercetin derivatives. A first peak at *m/z* 611 corresponding with a loss of a rutinose (*m/z* 308) was identified as quercetin-3-*O*-rutinoside. Two peaks with an *m/z* of 465 were identified as Quercetin-3-*O*-galactoside and quercetin-3-*O*-glucoside. Another peak at *m/z* 479, compatible with the loss of a glucuronide (m/176), was identified as quercetin-3-*O*-glucuronide. Finally, three peaks with an *m/z* of 435 and releasing a unique MS fragment at *m/z* 303, which corresponded to a loss of a pentose, were tentatively assigned as quercetin pentosides. The presence of the aglycone quercetin was also identified and confirmed with a commercial standard. The last peak, with a molecular ion at *m/z* 449 and MS fragment at m/ z 287, was identified as kaempferol-3-*O*-glucoside.

In the present extract, epicatechin was the only flavanol detected and this was confirmed with the commercial extract.

In the chromatographic conditions used in this study we detected some major peaks that were identified as some free amino acids (AAs) containing aromatic groups such as tryptophan and phenylalanine. The identification and quantification of these essential AAs was performed with commercial standards and is reported also in Table 1.

### 2.3. Cell Viability

In our study, it was found that the pretreatment of differentiated SH-SY5Y cells (by treatment with 10 µM RA) with up to 1 mg/mL of RB and up to 100 µM of (−) -epicatechin, catechin and cyanidin-3-glucoside did not have a significant effect on cell viability. Thus, it was ruled out that the effects found were not due to a cytotoxic effect.

### 2.4. Protective Effect of Polyphenols against Reactive Oxigen Species (ROS) Production

To determine the protective effects of RB extract against ROS production, a fluorescent probe mediated assay was performed by flow cytometry. In our conditions, the protective effect is associated with the inhibition of ROS formation that is induced by t-BHP in differentiated SH-SY5Y cells. T-BHP causes cell damage producing the increase in ROS formation and thus the damage of the different cell macromolecules. In order to optimize our working conditions, we established the working concentration by performing a dose response curve (data not shown). After exposing the cells to 500 µM t-BHP for 1 h, the maximum amount of ROS is produced and thus this treatment represents our activated control.

**Figure 1:**
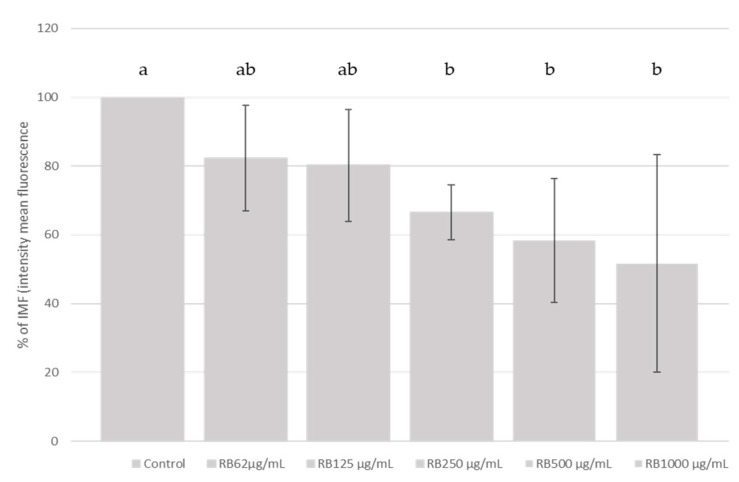
Measurement of ROS generation in SH-SY5Y differentiated cells after RB extract treatment. Protective effect of RB on total ROS generation induced by t-BHP treatment. SH-SY5Y differentiated cells were treated with increasing concentrations (1000, 500, 250, 125 and 62 µM) of RB for 3 h and the ROS generation was measured using the DCFH-DA assay. Figure shows the fold change in the percentage of mean fluorescence intensity as compared to the group treated with 500 µM t-BHP (maximum ROS production). Data represents the mean ± standard deviation of three independent experiments; different letters (a, b) indicate significant differences (*p* < 0.05). Linear effect of increasing level of RB on ROS generation.

The addition of RB linearly (*p* < 0.05) inhibited the ROS production generated by the addition of t-BHP in this SH-SY5Y differentiated cell model. When the RB treated cells were compared with the control group, the differentiated cells, pre-treated with 1000, 500 and 250 µg/mL of RB extract, reduced (*p* < 0.05) the ROS production by 48.3%, 41.7% and 33.5%, respectively (Figure 1).

**Figure 2:**
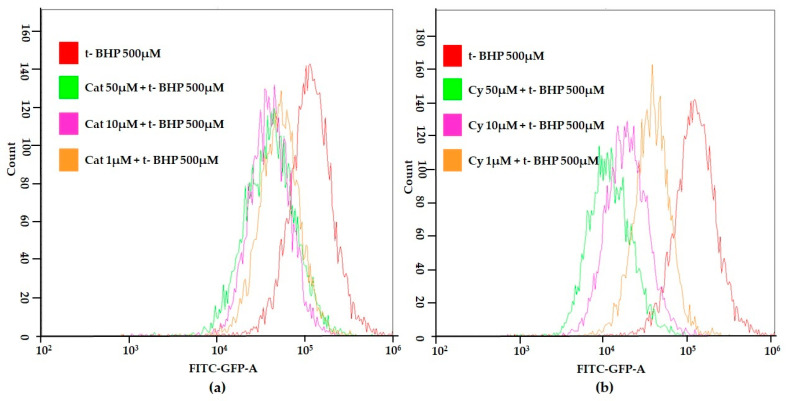
Measurement of ROS generation in SH-SY5Y differentiated cells after catechin (**a**) or cyanidin-3O-glucoside (**b**) treatment at different concentrations. SH-SY5Y differentiated cells were treated with increasing concentrations of either catechin (**a**) or cyanidin-3*O*-glucoside (**b**) for 3 h and the ROS generation was measured using the DCFH-DA assay.

Furthermore, the effects of epicatechin, catechin and cyanidin-3-*O*-glucoside on t-BHP induced ROS generation were investigated. As shown in Figure 2, as an example, the anthocyanin cyandin-3-*O*-glucoside and catechin at increased concentrations was able to decrease the signal corresponding to the maximum ROS formation generated by cell treatment with t-BHP in our conditions.

Flow cytometric histograms showing an increased fluorescence in the FL1-H channel indicate increased levels of ROS. As expected, the mean fluorescence intensity of the oxidized dichlorofluorescein (DCF) was increased compared to the controls after 500 μM t-BHP treatment in differentiated SH-SY5Y cells (red line). Our results showed that treatment with catechin (Figure 2a) and cyanidin-3-*O*-glucoside (Figure 2b) inhibited t-BHP-induced ROS production in differentiated SH-SY5Y cells in a dose-dependent manner.

**Figure 3:**
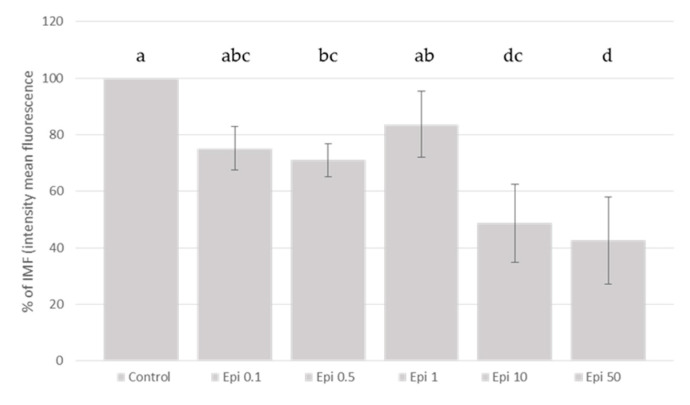
Protective effect of (−)-epicatechin on total ROS generation induced by t-BHP treatment. SH-SY5Y differentiated cells were treated with increasing concentrations of (−)-epicatechin (from 0.1 to 50 µM) for 3 h and the ROS generation was measured using the DCFH-DA assay. Data represent the mean ± standard deviation of three independent experiments. Different letters (a, b, c, d) indicate significant differences (*p* < 0.05).

**Figure 4:**
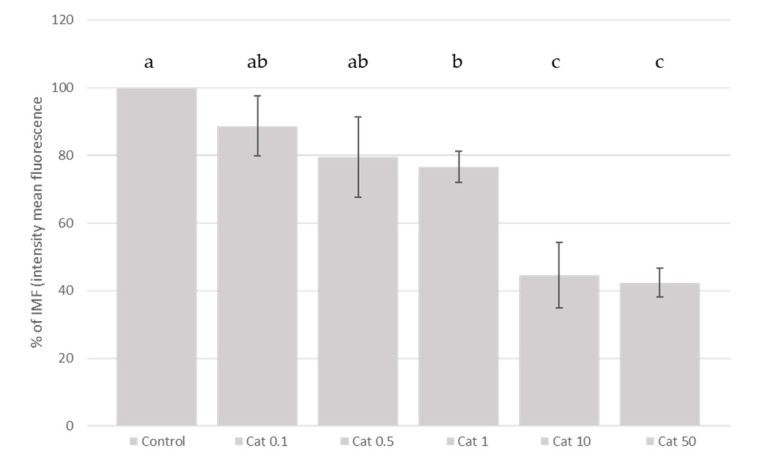
Measurement of ROS generation in SH-SY5Y differentiated cells after catechin treatment. SH-SY5Y differentiated cells were treated with increasing concentrations of catechin (from 0.1 to 50 µM) for 3 h and the ROS generation was measured using the DCFH-DA assay. Figure shows the fold change in the percentage of mean fluorescence intensity as compared to the group treated with 500 µM t-BHP (maximum ROS production). Data represent the mean ± standard deviation of three independent experiments. Different letters (a, b, c) indicate significant differences (*p* < 0.05).

**Figure 5:**
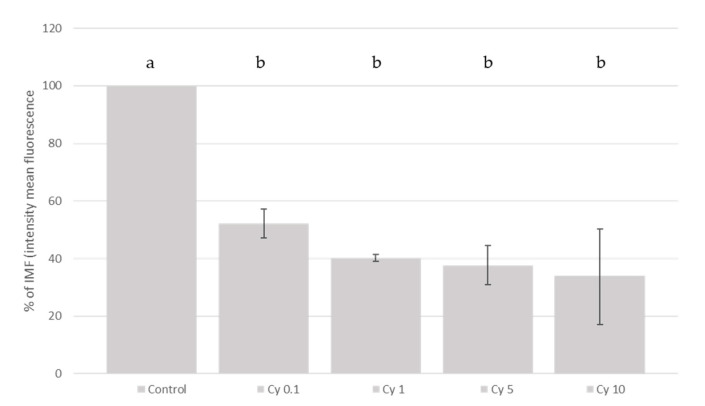
Measurement of ROS generation in SH-SY5Y differentiated cells after cyanidin-3-glucoside treatment. Protective effect of cyanidin on total ROS generation induced by t-BHP treatment. SH-SY5Y differentiated cells were treated with decreasing concentrations of cyanidin-3-glucoside (from 10 to 0.1 µM) for 3 h and the ROS generation was measured using the DCFH-DA assay. Figure shows the fold change in the percentage of mean fluorescence intensity as compared to the group treated with 500 µM t-BHP (maximum ROS production). Data represent the mean ± standard deviation of three independent experiments. Different letters (a, b) indicate significant differences (*p* < 0.05).

In general, the addition of the different polyphenols studied, linearly (*p* < 0.05) inhibited the ROS production generated by the addition of t-BHP in SH-SY5Y differentiated cells. Our results showed (Figure 3, Figure 4 and Figure 5) that the addition of cyanidin-3-*O*-glucoside, catechin and epicatechin linearly (*p* < 0.05) inhibited the ROS production generated with the t-BHP in this SH-SY5Y differentiated cell model. Regarding epicatechin (Figure 3), a reduction (*p* < 0.05) of ROS production was observed with concentrations of 50 and 10 µM (57.4% and 51.3%, respectively, compared with the control group). In the case of catechin, the ROS inhibition was observed at 50, 10 and 1 µM (57.7%, 55.4% and 23.4%, respectively, compared with the control group). The different cyanidin-3-*O*-glucoside concentrations studied (from 10 to 0.1 µM) reduced the ROS production (Figure 4).

## 3. Discussion

### 3.1. Compositional Characterization

Red berries contain a wide diversity of phenolic compounds including phenolic acids and flavonoids, anthocyanins and flavonols almost exclusively present in their glycosylated forms [24]. Among them, anthocyanins are water-soluble pigments that confer the blue, purple, and red color to the berries.

Delphinidin, cyanidin and peonidin derivatives, that constituted 46%, 37% and 18% of total anthocyanins, respectively, were the anthocyanins present in the red-berry extract. Delphinidin derivatives are the predominant anthocyanins present in blackcurrant cultivars and in some varieties of blueberry conferring to these fruits their dark blue or almost black color [24,26,27]. Cyanidin derivatives are the most commonly anthocyanin found in berries [28] and are responsible for the intense red color of their fruits. Peonidin derivatives are anthocyanins characteristic of blackcurrant and blueberry fruits [27]. The ability of anthocyanins to cross the BBB has been reported by Andrés la Cueva et al. (2005) [13]. These authors identified the presence of several anthocyanins (among them cyanidin-3-*O*-glucoside and galactoside, peonidin-3-*O*-arabinose and delphinidin-3-galactosides) in the brain of rats fed blueberries, suggesting their ability to cross the blood–brain barrier and affect learning activity and memory [13].

The sugar moiety is a determinant factor affecting the quantity and site of absorption of dietary flavonoid glycosides [29]. Thus, whereas glucoside derivatives are preferentially absorbed at the small intestine, the deglycosylation of rutinosides is limited to the colon where bacterial rhamnosidases catalyze the cleavage of rhamnose [30,31]. Rutinoside anthocyanins were the prevailing forms (accounting for 59% of total anthocyanins) identified in the berry extract used in this study followed by glucoside/galactosides and arabinoside anthocyanins (36% and 5%, respectively).

Regarding the flavonols, the use of standards (quercetin and quercetin-3-*O*-glucoside) along with the information from the MS fragmentation reported in the literature [23,25] allowed the identification of the different flavonol structures that are described in the Results section and Table 1. Different derivatives of the three flavonols quercetin, myricetin and kaempferol were detected and quantified (representing 49%, 49% and 2%, respectively) in the RB extract. These flavonols are commonly found in most fruits and vegetables and, as occurred for anthocyanins in the RB tested, were mainly found as 3-*O*-rutinosides and 3-*O*-glucosides (35% and 60% of total flavonols, respectively), and to a lesser extent as 3-*O*-arabinoside (5%), which is consistent with the literature [24,27].

The presence of monomeric, including epigallocatechin and oligomeric flavan-3-ols (most commonly B-type dimers), has been reported in blackcurrants, raspberries and blueberries [32,33]. However, in the present extract, epicatechin was the only flavanol detected. In this sense, we consider that the fact that the extraction was done with pure water in order to mimic physiological conditions, could have affected the composition of the final extract. In this sense, the acetone/water/acid system has been demonstrated to be the best solvent system to extract proanthocyanidins [34]; however, we decided to use water because the final aim of this work was to determine the effect of the RB extract on neuronal viability and redox response in conditions that could mimic physiological conditions. Another factor that could have influenced the composition in flava-3-ols is the method used in order to dry the RB. There are various factors that can influence the flavanol content in food products, from the variety to the extraction method through the agronomical conditions or the method used for fruit drying. Processing conditions and temperatures used during the drying procedure might affect the polyphenol stability [22]. However, since the berries were freeze-dried we consider that the effect is not due to the processing method in this case.

In the chromatographic conditions used in this study we identified two free amino acids (AAs) containing aromatic groups corresponding to tryptophan and phenylalanine. These AAs, precursors of many constituents of plants including phenolic compounds, are also precursors of neurotransmitters in animals. They contribute to the regulation of numerous physiological mechanisms and are involved in neuropsychiatric conditions characterized by acute or chronic inflammation [35].

### 3.2. Effect of RB and Polyphenols on Cell Viability and ROS

Scientific evidence suggests the capacity of polyphenols to reduce the ROS generation in neuronal cellular models [36,37,38]. In our study, we used t-BHP treatment to produce mitochondrial injury, which has been widely used to induce oxidative stress in the human neuroblastoma and in differentiated SH-SY5Y cells [39,40]. Multiple assays have been utilized to measure ROS production [41]. Although widely used, fluorescent measurement of ROS is less specific and in some cases does not take into consideration the cytotoxicity, while flow cytometry has been proven to be a more reproducible method to measure ROS generation [42]. In order to obtain the most accurate results for translation and comparison to in vivo models, we tested our product in a model including differentiated SH-SY5Y cells. Fully differentiated SH-SY5Y neurons provide a closer approximation of mature human neurons found in vivo than their undifferentiated progenitor cell counterparts, providing an advantageous model for chemotherapeutic toxicity in neurons [43].

In the present study, the addition of the red-berry extract linearly inhibited the ROS production generated with the t-BHP. Similar neuroprotective potential has been previously reported with the exposure to food extracts and single compounds in similar cell models but in undifferentiated cells. In this sense, the addition of red raspberry inhibited the apoptosis induction and ROS accumulation by enhancing the activity of catalase in H_2_O_2_-treated SH-SY5Y neuroblastoma cells [44]. The addition of blueberry and cranberry increased mitochondrial activity and reduced intracellular ROS production and lipid peroxidation induced by H_2_O_2_ [38]. These authors reported a similar protective effect when cyanidin-3-O-glucoside was added. The neuroprotective effects of cyanidin and cyanidin 3-O-glucoside has also been reported by other authors in neuroblastoma human cells [45,46]. In the present study, we observed that the addition of cyanidin linearly inhibited the ROS production generated with the t-BHP, demonstrating that the neuroprotective effect of cyanidin-3-glucoside was also reflected in differentiated neuronal cells. To the best of our knowledge, this is the first study reporting the effect of this anthocyanins in differentiated neuronal cells.

In SH-SY5Y cells, (+)-catechin, (−)-catechin or (⫾)-catechin reduced apoptosis induced by MPP+ and decreased ROS generation caused by MPP+. Different enantiomers of catechin showed protective effects at similar potency.

Different enantiomers of catechin and epicatechin showed similar protective effects in the SH-SY5Y neuroblastoma cell line [47,48,49]. However, their effectiveness in the differentiated cells had not been previously reported. Comparing the exposure to the single compounds, the protective effect seems to be more pronounced in the case of cyanidin-3-glucoside than that of epicatechin and catechin. In this sense, cyanidin-3-glucoside reduced ROS production when it was added to the media with a dose as low as 0.1 µM. On the other hand, both catechin and epicatechin showed a protective effect when assayed at concentrations higher than 10 µM. Moreover, in the case of catechin, the protective effect was already observed with a concentration of 1 µM. In this sense, cyanidin-3-glucoside (and possibly the rest of the anthocyanins present in the extract) seem to be responsible for the ROS inhibitory effect of RB extract. Our results might encourage further research in animal models of neurological diseases to explore the potential neuroprotective protection of anthocyanin-rich diets. 

## 4. Materials and Methods

### 4.1. Chemicals

All chemicals used were of HPLC analytical grade. Caffeic acid, 5-*O*-caffeoylquinic acid, p-coumaric acid, cyanidin-3-*O*- glucoside, quercetin-3-*O*-glucoside, l-phenylalanine and l-tryptophan were purchased from Sigma Chemical Co (St. Louis, MO, USA).

### 4.2. Test Product

A commercial freeze-dried red-berry mixture was kindly supplied by Salengei^®^ (Barcelona, Spain). The mixture contained a blend of red and black currant (16.7% and 33.3%, respectively), raspberry (33%) and blueberry (16.7%) and has been previously used in two human trials as one of the assayed foods (one unpublished and the other [50]).

### 4.3. Polyphenol Extraction

For the extraction of RB phenolic compounds, 5 g of sample were placed in a capped centrifuge tube and suspended in 20 mL of distilled water vortexed and sonicated during 15 min. Samples were then centrifuged at 5000 rpm for 10 min at 4 °C and the supernatant was collected. The residue was re-suspended in 20 mL of water and re-extracted following the same procedure twice. The supernatants were combined and freeze dried until their use. The RB water soluble extract was then used for cell culture assays. For the polyphenolic characterization, this RB extract was re-suspended in MeOH/H2O (50:50 *v*/*v* acidified with formic acid 0.1%), filtered (0.45 μm), and placed in vials for subsequent HPLC-QTOF-MSMS analysis of phenolic compounds.

### 4.4. HPLC-QTOF-MS Analysis of Phenolic Compounds

Analyses were performed using HPLC coupled with a mass spectrometer (HPLC-MS- QTOF). The HPLC (Agilent 1200, Agilent Technologies, Santa Clara, CA, USA) with a quaternary pump (G1311A) was coupled with a diode array detector (Agilent G1315B) and an Agilent 6530 Accurate-Mass QTOF LC /MS with Electrospray Ionization (ESI) and Jet Stream technology (Agilent Technologies). Separation was performed on a Phenomenex Luna C18 column (5 μm, 4.6 mm × 150 mm), set thermostatically at 25 °C.

A gradient between solvent A (water/formic acid, 99.9:0.1, *v*/*v*) and solvent B (acetonitrile/formic acid, 99.9:0.1, *v*/*v*) was applied at a flow rate of 0.5 mL/min as follows: 10% B at 0 min, 30% B at 30 min, 35% B at 35 min, 40% B at 45 min, 10% B at 50 min, and 10% B at 60 min. The volume of sample injected was 20 μL. The electrospray ionization (ESI) parameters were as follows: drying gas flow, 8 L/min; nebulizer pressure, 45 psi; gas drying temperature, 325 °C; sheath gas temperature, 300 °C; sheath gas flow, 11 L/min; capillary voltage, 4000 kV; fragmentator, 120 V. The ESI was operated in positive and negative modes to provide extra certainty in the determination of the molecular masses, and quantification was performed in positive mode. Data were collected in extended dynamic range, 100−1200 *m/z*. For the identification and quantification of compounds, MS and tandem mass spectrometry fragmentation spectra (MSMS) experiments were performed and spectral signal at data were also acquired at 280, 320 and 520 nm. For MSMS experiments, quite generic collision energy of 20 V was used, as a compromise, to simplify development of the method and ensure good fragmentation of the majority of targeted compounds. Data acquisition and processing were performed with the Masshunter Data Acquisition B.05.01 and Masshunter Qualitative Analysis B.07.00 SP2 software. Compounds were identified by comparing mass spectra and retention time with the corresponding standard, if available. In the case of compounds for which standards were not available, identification was based on prediction of chemical formula from accurate ion mass measurement and confirmed by comparing MSMS with data provided by relevant literature references (see Table 1). The quantification was performed by interpolation into the calibration curve of the standard or structurally related compound used to quantify (equivalent) and expressed as µg per g of dry matter (DM) as follows: epicatechin for flavan-3-ols, cyanidin-3-O-glucoside for cyanidin and delphinidin derivatives, peonidin-3-O-glucoside for peonidin derivatives, and quercetin-3-O-glucoside for flavonols.

### 4.5. Cell Culture and Differentiation

The human cell line SH-SY5Y (neuroblastoma) was maintained in Dulbecco Modified Eagle Medium (DMEM) supplemented with 10% fetal bovine serum (FBS), 1% antibiotics (penicillin/streptomycin 5000 U/mL) and 1% non-essential amino acids. The SH-SY5Y cell line was kindly provided by Dr. Almeida form de University of Salamanca. Cells were grown in 75 cm^2^ tissue culture flask at 37 °C in a humidified atmosphere containing 5% CO_2_ until confluence. Cells were harvested with trypsin-EDTA. SH-SY5Y cells were seeded in 96-well plates for the cytotoxicity test and in 24-well plates for the ROS assay.

Cell differentiation was induced with 10 µM all-trans retinoid-acid (RA) (Sigma-Aldrich, St Louis, MO, USA) following the methodology [51,52]. After 24 h, medium was replaced with medium in which the FBS concentration was reduced to 1%, supplemented with 10 μM of RA and incubated for 5 days. At the days 2 and 4, differentiating media was changed and all the experiments are performed after 5 days of differentiation. Only cells between passages P7 to P15 were used.

### 4.6. Cell Viability Assays

The viability of cells was determined by MTT assay. SH-SY5Y cells were plated in 96-well plates (2 × 10^4^ cells/well), cultures for 24 h at 37 °C in 5% CO_2_ and were differentiated for 5 days with 10 µM RA. The differentiated neurons were treated with serially diluted concentration of catechin (cat), epicatechin (epi), cyanidin (cy) (50–0.1 µM in EtOH/DMSO (1:5 *v*/*v*) and red fruit extract (final concentration: 1, 0.5, 0.25, 0.125, 0.62 and 0 mg/mL) dissolved in serum-free DMEM. After 3 and 18 h of incubation 20 µL of a MTT solution (5 mg/mL in PBS) was added to each well and incubated for an additional 2 h at 37 °C in 5% CO_2_. Formazan crystals formed in the wells were solubilized in 200 µL of DMSO (Dimethyl sulfoxide). Absorbance was measured at 570 nm wavelength employing a microplate reader PowerWaveTM XS (BioTek Instruments, Inc., Winooski, VT, USA).

The assay was repeated with three independent experiments. The viability was calculated in comparison to control experiments in which a solvent control was added in place of polyphenols and that was used as 100% viable reference [53].

### 4.7. Evaluation of Reactive Oxygen Species (ROS) Generation

To determine the protective effect of FR extract and polyphenols (epi, cat and cy) on t-BHP-induced reactive oxygen species (ROS) generation, SH-SY5Y cells were seeded into 24-well plates at a density of 2 × 10^5^ cells/well and cells were differentiated for 5 days with 10 µM RA. After incubation with FR extract (final concentration: 1, 0.5, 0.25, 0.125, 0.62 and 0 mg/mL dissolved in serum-free DMEM) or polyphenols (cat, epi, cy (50–0.1 µM in EtOH/DMSO (1:5 *v*/*v*)) for 3 h, then cells were treated with t-BHP (500 µM) for 1 h. Total ROS in cells was measured by using 2′, 7′-dichlorodihydrofluorescein diacetate (H2DCFDA, Cat D6883, Sigma-Aldrich). H2DCFDA (1 µM) was added to the culture medium and incubated for 30 min. H2DCFDA is oxidized by ROS into the highly fluorescent 2′, 7′-dichlorodihydrofluorescein (DCF), and the produced CDF is proportional to ROS generation [38]. Excess H2DCFDA was removed by washing the cells twice with PBS. Labeled cells were trypsinized and resuspended in PBS and then analyzed using a flow cytometer (Cytoflex, Beckman Coulter). A minimum of 10,000 cells was analyzed per condition. Then, all data were compared to control samples (maximum ROS production) whose fluorescence was considered as 100.

### 4.8. Statistical Analysis

Statistical data analysis was performed though SAS (Version 9.2, SAS Inst. Inc., Cary, NC, USA). Data were analyzed as a completely randomized block design using the general linear model procedure, where the compound studied (red fruit, epicatechin, catechin and cyanidin) was the main effect, and the experiment was used as a block effect. Additionally, a regression analysis using the proc-reg procedure was included to determine the linear and quadratic effects. Variance homogeneity was performed using Levene’s test. When the effect was considered significant (*p* < 0.05), means were compared using the Tukey’s range test. All analyses were performed in triplicate, and the results were displayed as average ± standard deviation.

## 5. Conclusions

In our experimental conditions, the addition of the red-berry extract, and the different polyphenols studied, inhibited ROS production generated with the t-BHP in SH-SY5Y differentiated cells, demonstrating their neuroprotective effect. Cyanidin-3-glucoside showed a more potent effect against ROS formation in SH-SY5Y differentiated cells than catechin and epicatechin, suggesting that cyanidin-3-glucoside (and possibly the rest of the anthocyanins present in the extract) are responsible for its ROS inhibitory effect. The present findings might serve as a first approach to recommend the use of this or a similar mixture of red berries as a preventive strategy to alleviate oxidative stress conditions in neuronal cells. The potential protective effect of red berries against neurodegenerative disease is mediated by a reduction of ROS levels and the protection of neuronal cells against the cytotoxicity caused by oxidative agents.

## Data Availability

The data presented in this study are available in article.
